# Efficacy of low‐fat milk and yogurt fortified with encapsulated vitamin D_3_ on improvement in symptoms of insomnia and quality of life: Evidence from the SUVINA trial

**DOI:** 10.1002/fsn3.1750

**Published:** 2020-07-06

**Authors:** Payam Sharifan, Mahdieh Khoshakhlagh, Zahra Khorasanchi, Susan Darroudi, Mitra Rezaie, Mohammad Safarian, Hassan Vatanparast, Asma Afshari, Gordon Ferns, Hamideh Ghazizadeh, Majid Ghayour Mobarhan

**Affiliations:** ^1^ Department of Nutrition School of Medicine Mashhad University of Medical Sciences Mashhad Iran; ^2^ Department of Medical Biochemistry Faculty of Medicine Mashhad University of Medical Sciences Mashhad Iran; ^3^ Metabolic Syndrome Research Center Mashhad University of Medical Sciences Mashhad Iran; ^4^ College of Pharmacy and Nutrition University of Saskatchewan Saskatoon SK Canada; ^5^ Brighton and Sussex Medical School Division of Medical Education Brighton UK; ^6^ International UNESCO center for Health‐Related Basic Sciences and Human Nutrition Mashhad University of Medical Sciences Mashhad Iran

**Keywords:** fortification, insomnia, sleep disorders, vitamin D_3_

## Abstract

**Introduction:**

Sleep disorders are a common condition globally. Vitamin D receptors are present on cells in several regions of the brain. It is possible that vitamin D status may affect brain function, including sleep patterns. We aimed to evaluate the 1,500 IU of Nano‐encapsulated vitamin D fortified in dairy products on the symptoms of insomnia and associated improvement of quality of life.

**Methods:**

A case series was undertaken as part of the *Survey of ultraviolent intake by nutritional approach* project. Subjects enrolled among adults with abdominal obesity. Twenty‐nine subjects with insomnia were selected according to the results of Insomnia Severity Index questionnaire and quality of life using a Short Form Health Survey (SF‐36) questionnaire. Subjects were allocated to four groups: low‐fat milk fortified by 1,500 IU vitamin D_3_ (*n* = 8), simple milk (*n* = 8), low‐fat yogurt fortified by 1,500 IU vitamin D_3_ (*n* = 7), and simple yogurt (*n* = 6) and were treated for 10 weeks.

**Results:**

The insomnia score improved after the intervention in the group receiving vitamin D fortified milk compared to group receiving unfortified milk (*p* < .001). There were no significant differences between the two groups taking yogurt (fortified vs. unfortified). Comparison of quality of life scores between baseline and after intervention indicated significant improvements in both fortified and simple milk groups (*p* = .002 and *p* = .03, respectively); but no differences were found in the groups taking yogurt.

**Conclusion:**

Fortified low‐fat milk containing 1,500 IU vitamin D_3_ can improve insomnia symptoms and subsequently quality of life.

Trial registration number: IRCT20101130005280N27, www.IRCT.ir.

## Graphical abstract

We aimed to evaluate the 1,500 IU of Nano‐encapsulated vitamin D fortified in dairy products on the symptoms of insomnia and associated improvement of quality of life. The insomnia score improved after the intervention in the group receiving Vitamin D fortified milk compared to group receiving unfortified milk (*p* < .001).There were no significant differences between the two groups taking yogurt (fortified vs. unfortified).

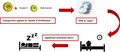

## 1 INTRODUCTION

The prevalence of sleep disorders has become a common challenge and approximately 10% of adult population in western countries have chronic insomnia (Ohayon, 2002). However, reports from other populations such as Iran have revealed that 50% of elderly who live in private home and 70% to 88.4% of residents in nursing home suffers from sleep disorders (Daglar, Pinar, Sabanciogullari, & Kav, 2014; Eser, Khorshid, & Cinar, 2007; Mousavi, Tavabi, Iran‐Pour, Tabatabaei, & Golestan, 2012). Insomnia is defined as difficulty in sleep maintenance, early morning waking, and unsatisfactory sleep quality (Ancoli‐Israel, Poceta, Stepnowsky, Martin, & Gehrman, 1997). The increased use of television and the Internet appears to have affected sleep patterns. Emerging data has revealed that a healthy life becomes at risk as a consequence of excessive or lack of sleep, and this may predispose to disorders such as type II diabetes, hypertension, metabolic disorders, and even increased mortality (Boucher, 2011; Kim et al., 2009; Kripke, 2003; Pigeon et al., 2008; Taylor, Lichstein, Durrence, Reidel, & Bush, 2005). Moderate sleep or one‐third part of the lifetime has optimal effect in daily routine. According to the National Sleep Foundation, adults should sleep around 7–8 hr per day; however, they may be different in terms of age and sex (Hirshkowitz et al., 2015). Healthy sleep is essential to preserve an appropriate balance between physical and mental health. The mechanism of daily sleep and wake is regulated by a physiological clock, a variety of neurons, and hormones produced by the central nervous system and environmental signals (Van Cauter et al., 2007). Recent literature supports the role of vitamin D in sleep disturbances. Vitamin D receptors (VDR) are located in cells of the anterior and posterior hypothalamus, substantia nigra, midbrain, raphe nuclei, and nucleus reticularis pontis oralis and caudalis. These are responsible for initiation and maintenance of sleep. Also pacemaker cells located in brainstem are supposed to have a significant role in the timing of the sleep. The probable effect of vitamin D in these brain areas suggests a preventable and treatable way for sleep disorders by sufficient intake of vitamin D.

Vitamin D as a fat‐soluble vitamin can be acquired from ultraviolet‐B radiation synthesized in the skin, dietary sources, various supplementations, and fortified foods (DRI, 2005). Vitamin D insufficiency and deficiency are prevalent globally (Palacios & Gonzalez, 2014). Although it has been estimated that 80% of vitamin D requirements is potentially fulfilled by exposure to ultraviolet light (Pilz et al., 2018), barriers such as having sedentary and less outdoor lifestyles, excessive use of sunscreen products, air pollutions, and style of clothing cause an increment of deficiency in populations; on the other hand, diets with high contents of vitamin D such as oily fish, fish liver oil, and wild mushrooms which are not a common part of usual intakes of several countries have made this inadequacy as a burden (Roth et al., 2018). In this regard, several strategies such as supplementation and fortification of vitamin D have been planned to eradicate or at least control vitamin D status (Pilz et al., 2018). Previously, consumption of milk and milk products has been indicated as a helpful method to improve sleep patterns which historically have been used especially in western populations before bedtime. Dairy products contain nutrients especially tryptophan as a substrate for production of serotonin and subsequently melatonin, which are play a significant function in sleep improvement (Peuhkuri, Sihvola, & Korpela, 2012). Although previous studies have shown the effects of vitamin D supplementation in sleep disorders, there is a lack of data regarding to efficacy of vitamin D as a fortified component in dairy products.

In this study, we aimed to evaluate the efficacy of low‐fat milk and yogurt fortified by 1,500 IU Nano encapsulated vitamin D in reduction of insomnia symptoms and subsequently improvement of quality of life in 10 weeks trial.

## 2 METHODS

### 2.1 Study design

This report as a pilot study is a part of *Survey of ultraviolent intake by nutritional approach* (SUVINA) study (trial registration: IRCT20101130005280N27, www.IRCT.ir) which was a triple‐phase study regarding the development of effective Nano encapsulated vitamin D_3_, for use in dairy products for assessment of stability and organoleptic characteristics, selection of best products for fortification by 1,500 IU vitamin D_3_ and evaluation of efficacy of vitamin D on physical and mental aspects of health in abdominal obese adults as a clinical trial.

This multicenter current study was conducted as a 10 weeks parallel blind randomized controlled clinical trial in Mashhad‐Iran between January 2019 and March 2019. Prior to data assembly, the study protocol was approved by Ethics Committee of the National Institute for Medical Research Development (protocol ID: IR.NIMAD.REC.1396.027). Participants gave written informed written consent prior to the start of the trial.

### 2.2 Participants

Subjects enrolled were among staff and students of Mashhad University of Medical Sciences who met the eligibility criteria. We recruited middle‐age adults (30–50 years) with abdominal obesity as a population sample of “potentially at risk” but “without chronic diseases” related to malignancies and liver or renal diseases (*n* = 306). Among 306 participants who had eligibility criteria, 289 participants finished the trial. Abdominal obesity was considered according to International Diabetes Federation as waist circumference ≥94 cm for men and ≥80 cm for women (Alberti, Zimmet, & Shaw, 2006). Other inclusion criteria were no intention or plan to change weight during the study, women who are not pregnant or lactating, no history of lactose intolerance or sensitivity, not using supplements containing vitamin D or any medications with interaction with vitamin D (corticosteroids, anticonvulsants, antidepressant, sleeping medications, etc.) in 3 months prior to the trial.

Exclusion criteria were based on willingness to continue participating at any time from the implementation of the study, pregnancy during the study, diagnosis of a disease or starting a specific treatment and occurrence of sensitivity or intolerance to dairy products.

Among all participants (*n* =289), we selected 29 subjects in different groups with insomnia symptoms according to the results of validated Insomnia Severity Index (ISI) questionnaire (Yazdi, Sadeghniiat‐Haghighi, Zohal, & Elmizadeh, 2012).

### 2.3 Randomization and blinding

Stratified block allocation was done for eligible subjects for center and sex status with ratio 1:1:1:1 to receive fortified low‐fat milk containing 1,500 IU Nano encapsulated vitamin D_3_/per serve (200 ml/day), simple low‐fat milk (200 ml/day) both for eight subjects, fortified low‐fat yogurt containing 1,500 IU Nano encapsulated vitamin D_3_/per serve (150 g/day) and simple low‐fat yogurt (150 g/day) for seven and six subjects respectively for 10 weeks’ trial. We used sealed envelopes containing A or B labels for placebo and intervention groups, respectively. Envelopes were opened in order and in front of each participant. Allocation list remained secured by faculty of medicine, and there was no access for researchers until the end of the study.

Blinding was implemented for subjects, investigators, statistician, and staff who allocated subjects into the groups (total blinding).

### 2.4 Nano encapsulated formulation and dairy products manufacture

Ingredients which were used for generating nanocapsules were as follows: precirol as solid lipid, oleic acid as liquid lipid, vitamin D as bioactive fatty core, poloxamer 188 as surfactant and deionized water. All components were incorporated by homogenization with high tensile stress and ultrasound.

Fortification of low‐fat milk and yogurt was carried out in the *Salamat* pilot dairy product factory under considerations of faculty of food sciences and technology (Ferdowsi University of Mashhad). Nutritional information for each 100 g milk and yogurt included: 56 kcal, sugar free, protein 7 g fat 3 g, and trans fatty acids 0.04 g.

Delivery and consumption of products (intervention or placebo) were done on production day or the next day after.

### 2.5 Outcome measurements

The primary endpoint was changed in sleepiness symptoms using the ISI validated questionnaire (Yazdi et al., 2012) after the 10 weeks trial period.

The ISI is a short subjective tool for assessment of insomnia symptoms and its consequences. The ISI comprised of seven items evaluating sleep initiation, sleep maintenance, early morning awakening, influence daily activities, perceived importance of worsening influenced due to sleep disorders, concerns about sleep problems, and contentment with sleep patterns (Morin, 1993). Each item based on severity scored on 0–4 scale. By summing the seven ratings, a total score is generated from 0 to 28, and every negative change reveals improvement.

The secondary endpoint was quality of life which was assessed using the Short Form Health Survey (SF‐36) validated questionnaire which was designed to assess general quality of life (Montazeri, Goshtasebi, Vahdaninia, & Gandek, 2005). SF‐36 categorized into eight headings: physical functioning (10 items), limitations due to physical difficulties, pain related to body, perception of general health, liveliness, social activities, limitations due to emotional difficulties, and perception of mental health, and also perception of changes in general health during 1‐year interval as *health transition* (Ware & Sherbourne, 1992). Scores range from 0 to 100. Questions were equally weighted and every positive change demonstrates improvement.

### 2.6 Laboratory measurements

The collection of venous blood samples was performed after 12 hr of fasting. Samples are allowed to clot at room temperature for 30–60 min and then centrifuged at 2,000 g. Sera were retrieved and kept into fresh microtubes, then stored at −80°C. Serum levels of 25(OH)D were assessed using commercial ELISA kits (Pishgaman sanjesh‐Iran) and an Awareness/Stat Fax 2,100 analyzer.

### 2.7 Statistical analysis

Quantitative data were indicted as mean ± *SD*, while qualitative as number and percentage. We checked normality for each variable with Kolmogorove–Smirnov test and Q‐Q plot. Within group analyses were performed by paired sample *t* test according to changes from baseline. Between group analyses were performed using *t* test, Mann–Whitney *U* test, or the chi‐square test as appropriate. Intention to treat was performed for all patients allocated and completed study. *p* < .05 were considered as significant. Since this study was a part of a bigger project (SUVINA study), we included cases who had insomnia symptoms in the baseline.

## 3 RESULTS

Between January and March 2019 a time when sun exposure is at a minimal level in Iran, we screened 306 participants for the main study (SUVINA study); after 1‐week run‐in period, they were allocated to four groups and passed 10 weeks trial.

We excluded 17 cases for our analyses because: serum vitamin D level >30 ng/ml at baseline (*n* = 3), they were using supplements or multivitamins during the trial (*n* = 4), refusal to continue the study (*n* = 7), one case for getting pregnant, one case for having arrhythmia, and one case for having a travel. From 289 participants, we found 29 cases that had symptoms of insomnia and also completed 10 weeks’ trial.

The mean age among these 29 participants was 43.2 ± 6.59, and 58.6% were female. Mean serum vitamin D among participants was 15.54 ± 4.79 ng/ml. There were no differences in distribution of age, using dairy products, and lipid profile between the four groups (Table 1). Also, there were no differences in serum 25(OH) D levels and scores of insomnia and quality of life among the groups.

**TABLE 1:** Baseline features of study population

	Intervention	Control	*p*‐Value
Milk (*n* = 16)			
Age (years)	39.5 ± 6.23	44.5 ± 5.63	.11
Sex			
Male	1 (20%)	4 (80%)	.14
Female	7 (63.6%)	4 (36.4%)	
Insomnia score	18.5 ± 3.33	17.25 ± 2.37	.4
Quality of life	83.25 ± 14.48	80.5 ± 9.6	.66
Vitamin D (ng/ml)	15.03 ± 3.91	14.9 ± 7.34	.96
Total cholesterol (mg/dl)	204.5 ± 18.81	211.1 ± 48.01	.72
Triglyceride (mg/dl)	185 ± 59.96	222.63 ± 170.93	.56
HDL‐C (mg/dl)	50.25 ± 11.99	45.13 ± 9.47	.35
LDL‐C (mg/dl)	116.75 ± 21.49	116 ± 29.37	.94
Yogurt (*n* = 13)			
Age (years)	47.42 ± 6.8	41.5 ± 5.99	.12
Sex			
Male	4 (57.1%)	3 (42.8%)	
Female	3 (50%)	3 (50%)	
Insomnia score	17.57 ± 2.22	16.66 ± 1.36	.4
Quality of life	86.71 ± 9.49	89.4 ± 6.65	.6
Vitamin D (ng/ml)	15.82 ± 4.09	16.72 ± 2.96	.66
Total cholesterol (mg/dl)	219.86 ± 34.86	199.17 ± 42.64	.35
Triglyceride (mg/dl)	220.29 ± 91.83	180.83 ± 97.95	.46
HDL‐C (mg/dl)	46.71 ± 6.77	47.17 ± 5.19	.89
LDL‐C (mg/dl)	117.29 ± 31.48	111.17 ± 32.82	.73

### 3.1 Vitamin D status

**FIGURE 1:**
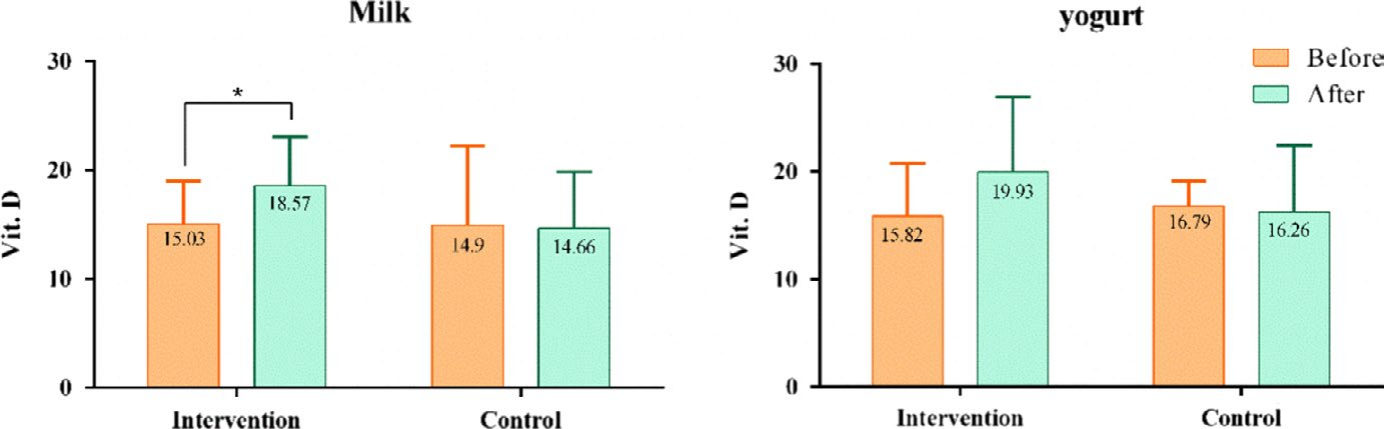
Serum 25(OH)D levels at baseline and after 10‐week intervention

At baseline, 85.9% of participants were vitamin D deficient and 13.4% were vitamin D insufficient. After the 10 weeks trial, serum 25(OH)D levels significantly improved in both fortified milk and fortified yogurt groups (*p* < .0001), but no sufficient levels (>30 ng/ml) were seen among participants (Figure 1). The percentage of deficient cases who met insufficiency criteria after the intervention were 16.2% and 28.2% in fortified milk and fortified yogurt, respectively.

### 3.2 Insomnia values

Insomnia score improved only in the group receiving fortified milk after intervention in comparison to group receiving unfortified milk (*p* < .001) while in group receiving yogurt (fortified compared with simple) no significant changes were seen (Table 2).

**TABLE 2:** Role of intervention in insomnia score and quality of life in the milk groups^a^

	Before intervention	After intervention	*p*‐Value
Milk (*n* = 16)			
Insomnia score			
Intervention	18.5 ± 3.33	13.62 ± 3.29	<.001
Control	17.25 ± 2.34	16.5 ± 4.02	.66
Quality of life			
Intervention	96.37 ± 10.9	79.37 ± 8.5	.002
Control	85.25 ± 10.48	80.5 ± 9.6	.03
Yogurt (*n* = 13)			
Insomnia score			
Intervention	13.28 ± 5.12	17.57 ± 13.28	.082
Control	13 ± 3.54	16.66 ± 1.36	.066
Quality of life			
Intervention	91.28 ± 10.84	84.42 ± 7.82	.205
Control	93.5 ± 12.51	86.5 ± 9.26	.2

^a^ Pair *t* test has been done.

### 3.3 Quality of life

Comparison of quality of life scores between baseline and after intervention indicated significant improvements in QoL in both fortified and unfortified milk groups (*p* = .002, *p* = .03, respectively); but no differences found in two other groups (Table 2).

## 4 DISCUSSION

This is the first parallel total‐blind RCT investigating the efficacy of vitamin D‐fortified milk and yoghurt in improvement of insomnia symptoms and quality of life among vitamin D deficient individuals with abdominal obesity. Our results showed that fortified low‐fat milk containing 1,500 IU Nano‐encapsulated vitamin D_3_/per serving (200 cc/day) resulted in a significant improvement in insomnia in the intervention group compared with the control group. Moreover, our results indicated a significant improvement in quality of life score in both vitamin D fortified and unfortified milk.

Earlier studies have shown effective role of vitamin D in regulating brain function. These studies have suggested that low serum concentration of 25(OH)D was associated with sleep disorders, depression, and impaired cognitive function (Balion et al., 2012; Milaneschi et al., 2014). One study demonstrated a negative correlation between low vitamin D concentration and insufficient sleep duration in premenopausal women (Darling, Skene, & Lanham‐New, 2011). Similarly, in a study by Jeong et al., it was found that inadequate sleep duration was positively associated with lower vitamin D levels in elderly Korean individuals (Kim, Chang, Kim, & Kang, 2014). Another study showed that supplementation with vitamin D increased sleep duration, improved sleep quality, and reduced sleep latency in individuals of 20–50 year‐old with sleep disorder (Majid, Ahmad, Bizhan, Hosein, & Mohammad, 2018). The results of some studies on the relation of vitamin D and sleep quality/disorders are inconsistent with our findings. For instance, Gunduz et al. conducted a study on pregnant women and observed no significant association between poor sleep quality and low vitamin D levels (Gunduz et al., 2016). Although the exact mechanisms to explain the association between insomnia and vitamin D are not yet understood, some mechanisms have been proposed. Previous experimental researches have reported that VDR are generally present in human brain, particularly the midbrain central gray, prefrontal cortex, hypothalamus, substantia nigra, and raphe nuclei, all of which are thought to play important role in sleep regulation (Gao et al., 2018). Moreover, inadequate vitamin D makes and expands myopathic pain, which in turn may cause poor sleep quality (Lee, Greenfield, & Campbell, 2009). Our results suggest that insomnia score improved only in the intervention group who received vitamin D‐fortified milk but not in the group who received vitamin D‐fortified yogurt. We suggested that the synergism effect of vitamin D with tryptophan in milk might be beneficial in reducing sleeping disturbances (Bakker‐Zierikzeea & Smitsb, 2007). It should be noted that tryptophan contents in simple milk based on USDA database are 0.043 mg/100 g compared to yogurt which is 0.02 mg/100 g. As the amount of tryptophan in milk is higher, the synergic effects of vitamin D and tryptophan can be explained by high amount of tryptophan. Although we could not find evidences to determine how much tryptophan is needed to alter levels of serotonin and subsequently melatonin for reduction of insomnia symptoms.

We found that after intervention, quality of life scores significantly improved in both vitamin D‐fortified and simple milks but no significant differences were observed in two other groups who received fortified and simple yoghurt. As mentioned earlier, vitamin D deficiency makes and develops muscle pain and strength, which may in turn cause poor sleep and reduced quality of life (Lee et al., 2009). A positive impact on sleep has been associated with tryptophan which is a precursor for serotonin synthesis that plays an important role in controlling sleep (Sarwar & Botting, 1999). In addition, studies have reported that milk contains various bioactive peptides, such as tryptic hydrolysate of αS1‐casein, containing a decapeptide αS1‐casein known as alpha‐casozepine (Guesdon et al., 2006). These peptides were shown to have anti‐stress effect and modulated anxiety. These effects of tryptic hydrolysate of αS1‐casein are thought to be mediated through its affinity to the gamma aminobutyric acid A receptors (dela Pena et al., 2016). In this reason, significant improved QoL in both groups of fortified and unfortified milk might be due to independent effects of bioactive components on other psychological factors such as anxiety and stress.

Several interventional studies have shown that vitamin D supplementation can be effective in improving quality of life (Costan, Vulpoi, & Mocanu, 2014; Gao et al., 2015), which are consistent with our findings. Huanget al. conducted a study on 28 veterans with multiple areas of chronic pain and low serum 25(OH)D concentration and observed that vitamin D supplementation might be effective in improving various aspects of quality of life, sleep, and pain (Huang, Shah, Long, Crankshaw, & Tangpricha, 2013). Costan et al. also reported that vitamin D‐fortified bread can improve quality of life in nursing home residents with vitamin D deficiency (Costan et al., 2014). In contrast, Matthews et al. observed no significant improvement in quality of life or depression symptoms following administration of vitamin D‐fortified milk (Matthews et al., 2019). The reasons for these controversial results may be due to differences in the health status of subjects, study duration, and vitamin D received as intervention. In addition, vitamin D‐fortified yogurt failed to improve the quality of life in the participants, which may be partly explained by the short duration of our trial.

Based on our knowledge, this is the first trial to examine the effects of vitamin D‐fortified milk and yoghurt on insomnia and quality of life in healthy subjects. The strengths of our analysis include the total blind randomized controlled clinical trial design, and using validated tools for assessment of insomnia and quality of life. Also using nanotechnology as a novel method in the vitamin fortification industry especially for fat‐soluble components made this study more qualified.

The limitations of this study include short‐term follow‐up, small sample size and the evaluation of quality of life and sleep which were based on self‐administered tools instead of more accurate face‐to‐face interviews.

## 5 CONCLUSION

In conclusion, we found that fortified low‐fat milk containing 1,500 IU Nano‐encapsulated vitamin D_3_/per serving (200 cc/day) can improve insomnia symptoms compared with the control group. Moreover, our results demonstrated significant improvements in quality of life score in both groups receiving fortified and unfortified milk subsequently to improvement in insomnia symptoms.

## CONFLICT OF INTEREST

The authors have no conflicts of interest to declare.

## References

[fsn31750-bib-0001] Alberti, K. G. M. M. , Zimmet, P. , & Shaw, J. (2006). Metabolic syndrome—A new world‐wide definition. A consensus statement from the international diabetes federation. Diabetic Medicine, 23(5), 469–480. 10.1111/j.1464-5491.2006.01858.x 16681555

[fsn31750-bib-0002] Ancoli‐Israel, S. , Poceta, J. S. , Stepnowsky, C. , Martin, J. , & Gehrman, P. (1997). Identification and treatment of sleep problems in the elderly. Sleep Medicine Reviews, 1(1), 3–17. 10.1016/S1087-0792(97)90002-2 15310520

[fsn31750-bib-0003] Bakker‐Zierikzeea, A. M. , & Smitsb, M. G. (2007). Sleep improving effects of milk. NSWO, 18, 29–32.

[fsn31750-bib-0004] Balion, C. , Griffith, L. E. , Strifler, L. , Henderson, M. , Patterson, C. , Heckman, G. , … Raina, P. (2012). Vitamin D, cognition, and dementia: A systematic review and meta‐analysis. Neurology, 79(13), 1397–1405. 10.1212/WNL.0b013e31826c197f 23008220 PMC3448747

[fsn31750-bib-0005] Boucher, J. (2011). insufficiency and diabetes risks. Current Drug Targets, 12(1), 61–87. 20795936 10.2174/138945011793591653

[fsn31750-bib-0006] Costan, A. R. , Vulpoi, C. , & Mocanu, V. (2014). Vitamin D fortified bread improves pain and physical function domains of quality of life in nursing home residents. Journal of Medicinal Food, 17(5), 625–631. 10.1089/jmf.2012.0210 24827747

[fsn31750-bib-0007] Daglar, G. , Pinar, S. E. , Sabanciogullari, S. , & Kav, S. (2014). Sleep quality in the elderly either living at home or in a nursing home. The Australian Journal of Advanced Nursing, 31(4), 6.

[fsn31750-bib-0008] Darling, A. , Skene, D. , & Lanham‐New, S. (2011). Preliminary evidence of an association between vitamin D status and self‐assessed sleep duration but not overall sleep quality: Results from the D‐FINES study of South Asian and Caucasian pre‐and post‐menopausal women living in Southern England. Proceedings of the Nutrition Society, 70 10.1017/S0029665111001285

[fsn31750-bib-0009] dela Peña, I. J. I. , Kim, H. J. , de la Peña, J. B. , Kim, M. , Botanas, C. J. , You, K. Y. , … Cheong, J. H. (2016). A tryptic hydrolysate from bovine milk αs1‐casein enhances pentobarbital‐induced sleep in mice via the GABAA receptor. Behavioural Brain Research, 313, 184–190. 10.1016/j.bbr.2016.07.013 27401107

[fsn31750-bib-0010] DRI . (2005). Institute of Medicine, Food and Nutrition Board, Dietary Reference Intakes: Energy, carbohydrate, fiber, fat, fatty acids, cholesterol, protein and amino acids. Washington, DC: The National Academy Washington.

[fsn31750-bib-0011] Eser, I. , Khorshid, L. , & Cinar, S. (2007). Sleep quality of older adults in nursing homes in Turkey: Enhancing the quality of sleep improves quality of life. Journal of Gerontological Nursing, 33(10), 42–49. 10.3928/00989134-20071001-07 17955737

[fsn31750-bib-0012] Gao, L.‐H. , Zhu, W.‐J. , Liu, Y.‐J. , Gu, J.‐M. , Zhang, Z.‐L. , Wang, O. , … Xu, L. (2015). Physical performance and life quality in postmenopausal women supplemented with vitamin D: A two‐year prospective study. Acta Pharmacologica Sinica, 36(9), 1065–1073. 10.1038/aps.2015.55 26279157 PMC4561972

[fsn31750-bib-0013] Gao, Q. , Kou, T. , Zhuang, B. , Ren, Y. , Dong, X. , & Wang, Q. (2018). The Association between vitamin D deficiency and sleep disorders: A systematic review and meta‐analysis. Nutrients, 10(10), 1395 10.3390/nu10101395 PMC6213953 30275418

[fsn31750-bib-0014] Guesdon, B. , Messaoudi, M. , Lefranc‐Millot, C. , Fromentin, G. , Tomé, D. , & Even, P. C. (2006). A tryptic hydrolysate from bovine milk αS1‐casein improves sleep in rats subjected to chronic mild stress. Peptides, 27(6), 1476–1482. 10.1016/j.peptides.2005.10.001 16303212

[fsn31750-bib-0015] Gunduz, S. , Kosger, H. , Aldemir, S. , Akcal, B. , Tevrizci, H. , Hizli, D. & Celik, H. T. (2016). Sleep deprivation in the last trimester of pregnancy and inadequate vitamin D: Is there a relationship? Journal of the Chinese Medical Association, 79(1), 34–38. 10.1016/j.jcma.2015.06.017 26391786

[fsn31750-bib-0016] Hirshkowitz, M. , Whiton, K. , Albert, S. M. , Alessi, C. , Bruni, O. , DonCarlos, L. … Adams Hillard, P. J. (2015). National Sleep Foundation’s sleep time duration recommendations: Methodology and results summary. Sleep Health, 1(1), 40–43. 10.1016/j.sleh.2014.12.010 29073412

[fsn31750-bib-0017] Huang, W. , Shah, S. , Long, Q. , Crankshaw, A. K. , & Tangpricha, V. (2013). Improvement of pain, sleep, and quality of life in chronic pain patients with vitamin D supplementation. The Clinical Journal of Pain, 29(4), 341–347. 10.1097/AJP.0b013e318255655d 22699141

[fsn31750-bib-0018] Kim, J. H. , Chang, J. H. , Kim, D. Y. , & Kang, J. W. (2014). Association between self‐reported sleep duration and serum vitamin D level in elderly Korean adults. Journal of the American Geriatrics Society, 62(12), 2327–2332. 10.1111/jgs.13148 25516029

[fsn31750-bib-0019] Kim, J.‐M. , Stewart, R. , Kim, S.‐W. , Yang, S.‐J. , Shin, I. , & Yoon, J.‐S. (2009). Insomnia, depression, and physical disorders in late life: A 2‐year longitudinal community study in Koreans. Sleep, 32(9), 1221–1228. 10.1093/sleep/32.9.1221 19750927 PMC2737580

[fsn31750-bib-0020] Kripke, D. F. (2003). Sleep and mortality. Psychosomatic Medicine, 65(1), 74 10.1097/01.PSY.0000039752.23250.69 12554817

[fsn31750-bib-0021] Lee, P. , Greenfield, J. R. , & Campbell, L. V. (2009). Vitamin D insufficiency—A novel mechanism of statin‐induced myalgia? Clinical Endocrinology, 71(1), 154–155. 10.1111/j.1365-2265.2008.03448.x 19178510

[fsn31750-bib-0022] Majid, M. S. , Ahmad, H. S. , Bizhan, H. , Hosein, H. Z. M. , & Mohammad, A. (2018). The effect of vitamin D supplement on the score and quality of sleep in 20–50 year‐old people with sleep disorders compared with control group. Nutritional Neuroscience, 21(7), 511–519. 10.1080/1028415X.2017.1317395 28475473

[fsn31750-bib-0023] Matthews, J. , Torres, S. J. , Milte, C. M. , Hopkins, I. , Kukuljan, S. , Nowson, C. A. & Daly, R. M. (2019). Effects of a multicomponent exercise program combined with calcium–vitamin D 3‐enriched milk on health‐related quality of life and depressive symptoms in older men: Secondary analysis of a randomized controlled trial. European Journal of Nutrition, 59(3), 1081–1091. 10.1007/s00394-019-01969-8 30993400

[fsn31750-bib-0024] Milaneschi, Y. , Hoogendijk, W. , Lips, P. , Heijboer, A. , Schoevers, R. , Van Hemert, A. , … Penninx, B. W. J. H. (2014). The association between low vitamin D and depressive disorders. Molecular Psychiatry, 19(4), 444 10.1038/mp.2013.36 23568194

[fsn31750-bib-0025] Montazeri, A. , Goshtasebi, A. , Vahdaninia, M. , & Gandek, B. (2005). The Short Form Health Survey (SF‐36): Translation and validation study of the Iranian version. Quality of Life Research, 14(3), 875–882. 10.1007/s11136-004-1014-5 16022079

[fsn31750-bib-0026] Morin, C. M. (1993). Insomnia: Psychological assessment and management, Vol. 281, (pp. 991–999). New York, NY: Guilford Press.

[fsn31750-bib-0027] Mousavi, F. , Tavabi, A. , Iran‐Pour, E. , Tabatabaei, R. , & Golestan, B. (2012). Prevalence and associated factors of insomnia syndrome in the elderly residing in Kahrizak nursing home, Tehran, Iran. Iranian Journal of Public Health, 41(1), 96. 23113128 PMC3481663

[fsn31750-bib-0028] Ohayon, M. M. (2002). Epidemiology of insomnia: What we know and what we still need to learn. Sleep Medicine Reviews, 6(2), 97–111. 10.1053/smrv.2002.0186 12531146

[fsn31750-bib-0029] Palacios, C. , & Gonzalez, L. (2014). Is vitamin D deficiency a major global public health problem? The Journal of Steroid Biochemistry and Molecular Biology, 144, 138–145. 10.1016/j.jsbmb.2013.11.003 24239505 PMC4018438

[fsn31750-bib-0030] Peuhkuri, K. , Sihvola, N. , & Korpela, R. (2012). Diet promotes sleep duration and quality. Nutrition Research, 32(5), 309–319. 10.1016/j.nutres.2012.03.009 22652369

[fsn31750-bib-0031] Pigeon, W. R. , Hegel, M. , Unützer, J. , Fan, M.‐Y. , Sateia, M. J. , Lyness, J. M. , … Perlis, M. L. (2008). Is insomnia a perpetuating factor for late‐life depression in the IMPACT cohort? Sleep, 31(4), 481–488. 10.1093/sleep/31.4.481 18457235 PMC2279755

[fsn31750-bib-0032] Pilz, S. , März, W. , Cashman, K. D. , Kiely, M. E. , Whiting, S. J. , Holick, M. F. , … Zittermann, A. (2018). Rationale and plan for vitamin D food fortification: A review and guidance paper. Frontiers in Endocrinology, 9, 373 10.3389/fendo.2018.00373 30065699 PMC6056629

[fsn31750-bib-0033] Roth, D. E. , Abrams, S. A. , Aloia, J. , Bergeron, G. , Bourassa, M. W. , Brown, K. H. , … Whiting, S. J. (2018). Global prevalence and disease burden of vitamin D deficiency: A roadmap for action in low‐and middle‐income countries. Annals of the New York Academy of Sciences, 1430(1), 44–79. 10.1111/nyas.13968 30225965 PMC7309365

[fsn31750-bib-0034] Sarwar, G. , & Botting, H. G. (1999). Liquid concentrates are lower in bioavailable tryptophan than powdered infant formulas, and tryptophan supplementation of formulas increases brain tryptophan and serotonin in rats. The Journal of Nutrition, 129(9), 1692–1697. 10.1093/jn/129.9.1692 10460206

[fsn31750-bib-0035] Taylor, D. J. , Lichstein, K. L. , Durrence, H. H. , Reidel, B. W. , & Bush, A. J. (2005). Epidemiology of insomnia, depression, and anxiety. Sleep, 28(11), 1457–1464. 10.1093/sleep/28.11.1457 16335332

[fsn31750-bib-0036] Van Cauter, E. , Holmbäck, U. , Knutson, K. , Leproult, R. , Miller, A. , Nedeltcheva, A. , … Spiegel, K. (2007). Impact of sleep and sleep loss on neuroendocrine and metabolic function. Hormone Research in Paediatrics, 67(Suppl. 1), 2–9. 10.1159/000097543 17308390

[fsn31750-bib-0037] Ware Jr., J. E. , & Sherbourne, C. D. (1992). The MOS 36‐item short‐form health survey (SF‐36): I. Conceptual framework and item selection. Medical Care, 30, 473–483. 1593914

[fsn31750-bib-0038] Yazdi, Z. , Sadeghniiat‐Haghighi, K. , Zohal, M. A. , & Elmizadeh, K. (2012). Validity and reliability of the Iranian version of the Insomnia Severity Index. The Malaysian Journal of Medical Sciences, 19(4), 31. 23613647 PMC3629678

